# A thin-film temperature sensor based on a flexible electrode and substrate

**DOI:** 10.1038/s41378-021-00271-0

**Published:** 2021-06-01

**Authors:** Zhaojun Liu, Bian Tian, Bingfei Zhang, Jiangjiang Liu, Zhongkai Zhang, Song Wang, Yunyun Luo, Libo Zhao, Peng Shi, Qijing Lin, Zhuangde Jiang

**Affiliations:** State Key Laboratory for Mechanical Manufacturing Systems Engineering, Xi’an Jiaotong University, 710049 Xi’an, China

**Keywords:** Physics, Electrical and electronic engineering

## Abstract

Accurate temperature measurements can efficiently solve numerous critical problems and provide key information. Herein, a flexible micro-three-dimensional sensor, with a combination of platinum and indium oxide to form thermocouples, is designed and fabricated by a microfabrication process to achieve in situ real-time temperature measurements. The stability and reliability of the sensor are greatly improved by optimizing the process parameters, structural design, and preparation methods. A novel micro-three-dimensional structure with better malleability is designed, which also takes advantage of the fast response of a two-dimensional thin film. The as-obtained flexible temperature sensor with excellent stability and reliability is expected to greatly contribute to the development of essential components in various emerging research fields, including bio-robot and healthcare systems. The model of the application sensor in a mask is further proposed and designed to realize the collection of health information, reducing the number of deaths caused by the lack of timely detection and treatment of patients.

## Introduction

Temperature is one of the most important physical parameters to measure in many fields. Based on decisions during the COVID-19 response in 2020, the human body temperature was tested accurately and conveniently in public places by a temperature measuring gun and thermal imager. Attached sensors are also feasible methods to realize the real-time monitoring of human body temperature. In recent years, sensors deposited on flexible substrates have been increasingly studied due to their excellent bending resistance, durability, and extensive application^1–6^. A thermocouple is a passive device used for measuring temperature. Compared with traditional bulk thermocouples, thin film thermocouples (TFTCs) have typical two-dimensional characteristics and possess the advantages of a small heat capacity, and fast response speed^5–9^. Moreover, TFTCs based on flexible substrates can meet the application requirements of real-time monitoring of the surface temperatures of nonplanar objects, especially complex geometric shapes with high curvature^10–15^.

To date, many innovative structures, along with new materials and different preparation techniques have emerged in the design and preparation of flexible temperature sensors^16–19^. Some researchers have even integrated or assembled flexible temperature sensors with other sensors to form an electronic skin (e-skin)^10,12–14,19^. The temperature sensors in e-skin are based on resistance thermometers, p–n junctions or composite materials undergoing thermal expansion^13,20–25^. In 2016, resistance thermometers with a linear temperature coefficient of resistance were used to measure the thermal properties of skin with a resolution of 0.014 °C. However, the sensitivity of the sensor was low, and the sensing mechanism was strain-sensitive; thus, its stretchability must be modified using buckling or rigid island approaches. In 2018, Hua et al.^26^ presented a skin-inspired highly stretchable and conformable matrix network that successfully expanded the sensing functionality of e-skin, including but not limited to temperature, in-plane strain, and pressure. Platinum was selected as the sensitive material, and a stretchable structure of platinum was designed to reduce the error caused by deformation during practical application. In 2020, Liu et al.^27^ prepared a flexible temperature sensor with an ultrahigh sensitivity based on the principle of thermoelectricity. In the actual test application on a 2-finger manipulator, the output voltage of the sensor showed two-step signals during service when the sensor touched and left the outer wall of the beaker. Although the flexible temperature sensor in the e-skin could detect the temperature of the measured object, the measurement error caused by contact deformation could not be neglected^28^.

To solve the above problems, various measures are proposed in this work. Platinum and indium oxide (In_2_O_3_) were chosen as the thermoelectrodes. As the most ductile metal, platinum cannot be damaged easily by external force and deformation. As a thermoelectric material, In_2_O_3_ has high temperature sensitivity. When combined, the matched work function can form an ohmic contact to form a thermocouple for measuring temperature. In terms of structure, the sensitive thin films are designed to have a bend-resistant structure, in which the surface of the flexible substrate is machined into a wavy shape so that the traditional two-dimensional flexible structure is transformed into a three-dimensional flexible structure^29^. Moreover, multiple methods to optimize the preparation process parameters are proposed. On the one hand, an orthogonal experiment is designed to optimize the sensing material. Among the samples, the thermoelectric electrode that has the least influence on the deformation of the heating electrode is selected as the preparation process parameter. On the other hand, the flexible substrate is forced into a prebent state before the thermal electrode is prepared by magnetron sputtering technology. The developed flexible temperature sensor has good deformation resistance and stability when measuring surfaces with different curvatures. Based on the ultrathin, ultralight, and ultraflexible characteristics of the prepared sensor, two models of smart masks are proposed. This sensor is expected to be used in human respiratory monitoring systems to realize big data collection of human health.

## Materials and methods

### Design of the sensor

Flexible TFTCs are composed of two parts: a temperature-sensitive layer and a flexible substrate. In this paper, in the temperature sensing layer, platinum and indium oxide were selected as the positive and negative electrodes of the thermocouple to improve the sensitivity of the sensor. However, the In_2_O_3_ material showed poor resistance to deformation and exhibited poor toughness. Polyimide was selected as the flexible substrate due to its outstanding comprehensive flexibility and strength, while also having good chemical resistance.

**Fig. 1:**
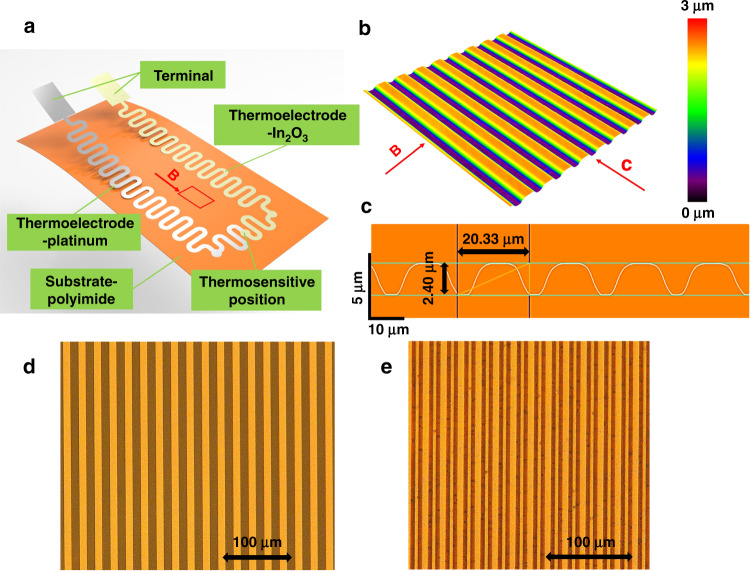
Structural diagram of the sensor. **a** Flexible micro-three-dimensional sensor for measuring temperature. **b** Microstructure of the substrate. **c** Cross-section of the substrate microstructure. **d** Lithographic mask and **e** polyimide substrate observed by optical microscopy.

A schematic diagram of the micro-three-dimensional flexible TFTCs is shown in Fig. 1a. To increase the resistance to deformation and reduce the internal stress caused by deformation, the two thermoelectrode patterns of the TFTCs aimed at measuring temperature were designed into curved shapes. The overlapping part of the thermoelectrodes at the upper end was the thermosensitive position for measuring temperature, which was a circle with a diameter of 2 mm. The width of the thermoelectrodes was 1 mm. At the terminal of the sensor, the two rectangles were used to connect wires for collecting the thermoelectromotive force (TEMF). Figure 1b, c show the confocal laser scanning microscopy images. As depicted in Fig. 1b, to further enhance the resistance of the sensor to deformation, a polyimide substrate was designed and prepared by a microfabrication process to obtain a wavy microstructure. Figure 1c shows the cross-section of the polyimide, from which we can observe that the polyimide substrate was prepared with a wavy microstructure that had a period of 20 μm and a depth of 2.4 μm.

### Preparation of the sensor

**Fig. 2:**
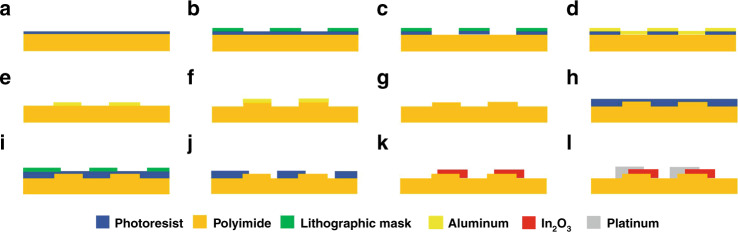
Preparation process of the sensor. **a**–**g** Fabrication of the wavy substrate and **h**–**l** preparation of the thermoelectrodes of the sensor.

As shown in Fig. 2, the preparation process of the sensor was mainly divided into two parts: the first part was the fabrication of the wavy substrate shown in Fig. 2a–g, and the second part was the preparation of the thermoelectrodes of the TFTCs exhibited in Fig. 2h–l. In Fig. 2a, the polyimide substrate was attached to a silicon wafer, and the photoresist was coated with a homogenizer onto polyimide. In Fig. 2b, c, the photoresist was exposed by a lithographic mask with a period of 20 μm. As shown in Fig. 1d, the lithographic mask was observed by optical microscopy. In Fig. 2d, magnetron sputtering technology was used to deposit aluminum, which was applied as a cover for the subsequent dry etching step. In Fig. 2e, the whole film was peeled off the remaining photoresist on the substrate using a lift-off process, thereby leaving behind the desired film pattern. In Fig. 2f, g, the surface of polyimide was etched with oxygen ions by the inductively coupled plasma (ICP) technique, and the remaining aluminum was removed with phosphoric acid. Thus, as shown in Fig. 1e, polyimide with a periodic microstructure was obtained. In Fig. 2h–l, magnetron sputtering technology was used to sequentially deposit the two thermoelectrodes of the sensor for measuring temperature, and the patterns were transferred to the substrate by using photolithography masks. The remaining photoresist on the substrate was removed using a lift-off process so that the desired pattern was left on the substrate.

### Simulation

Before preparing the sensor, mechanical and thermoelectric simulations were carried out with linear and curved TFTCs. AutoCAD and SolidWorks were used as the two-dimensional and three-dimensional models, respectively, and COMSOL was used for the further analysis of properties.

**Fig. 3:**
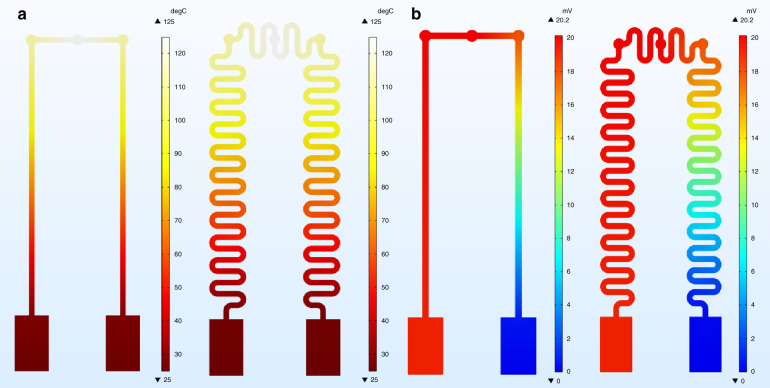
Comparison of the thermoelectric properties between the linear sensor and curved sensor. **a** Temperature distribution of the sensors and **b** TEMF distribution of the sensors.

In the thermoelectric simulation, the boundary conditions of the flexible sensors were set according to the actual test system and test conditions. The left thermoelectrode was platinum, and the right thermoelectrode was In_2_O_3_. The sensor terminal was set to 25 °C as the ambient temperature, and the thermal position was heated to 125 °C at different temperatures. Moreover, the terminus of the In_2_O_3_ thin film was grounded. Both structures exhibited the same static thermoelectric properties, including the temperature field and electric potential field. As shown in Fig. 3, the maximal TEMF was 20.2 mV, as measured by the probe at the terminal when the temperature difference was 100 °C between the thermosensitive position and terminal. The average Seebeck coefficient was approximately 0.202 mV/°C. The simulation results also revealed that the static thermoelectric characteristics of the flexible sensors were independent of the thickness and the structure of the thermoelectrodes. According to Eq. 1, which was used for calculating the TEMF of thermocouples, the simulation results were accurate and reliable, in which *α*_ab_ represents the TEMF rate of the two materials, *T* represents the temperature, *n* represents the carrier concentration of In_2_O_3_ at temperature *T*, *k*_0_ is the Boltzmann constant, *q* stands for the charge, *E*_F_ represents the Fermi levels of the metal, and *N* is the effective state density of the conduction band.

1 $$\alpha _{{\rm{ab}}} = \frac{{d({\rm{EMF}})}}{{dT}} = \alpha _{{\rm{metal}}} - \alpha _{{\rm{semiconductor}}} = - \frac{{T\pi ^2k_0^2}}{{qE_{\rm{F}}}} + \frac{{k_0}}{q}\left( {\frac{3}{2} - In\frac{n}{N}} \right)$$

The TEMF rate was only related to the carrier concentration of the material. Regarding semiconductors, the carrier concentration and diffusion method are directly affected by temperature. Therefore, for our TFTCs, the temperature gradient and material characteristics were the two vital factors that determined the value of the TEMF.

During the service of an e-skin, stretching and bending are the two most common deformations. In the mechanical simulation, according to the actual application state of the sensor, the internal stress fields of the two kinds of sensors under two kinds of deformation were simulated and compared. Since the platinum electrode had better ductility and toughness than the In_2_O_3_ electrode, it was not prone to fracture and damage during deformation. In practical application, the cracks in the In_2_O_3_ thermoelectrode would lead to sensor failure. Therefore, in the simulation, the sensitive layer material was set to indium oxide to more intuitively compare the mechanical properties of the two structures.

**Fig. 4:**
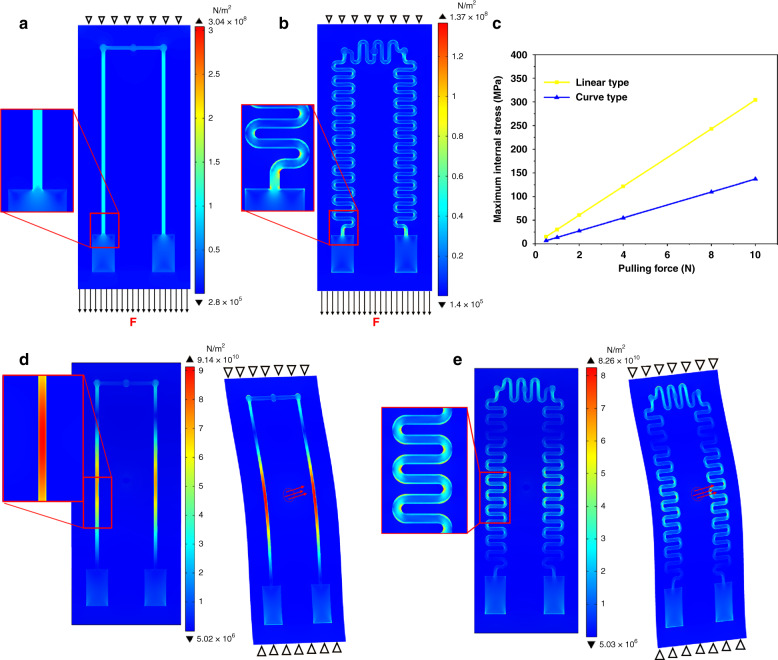
Comparison of the mechanical properties between the linear sensor and curved sensor. Internal stress distribution and a detailed diagram of the maximal stress of **a** the linear and **b** curved sensors under tensile stress. **c** Maximal internal stress variation of the two sensors under different uniformly distributed loads. The distribution of internal stress, detailed diagram of the maximum stress force and deformation diagrams of the **d** linear and **e** curved sensor under bending deformation.

During the simulation of tensile deformation, a fixed constraint was applied to the top surface of the substrate, and a uniform downward load from 0.5 N to 10 N was applied on the substrate surface. The distribution of internal stress under tensile deformation is shown in Fig. 4a–c. The maximum internal stress of both structures was generated in the thermoelectric materials, which were close to the terminal pads. Furthermore, under the same conditions, the curved sensor greatly reduced the internal stress generated in the structure. Compared with the linear sensor, the maximum internal stress was reduced to 45.07%. By optimizing the structure of the thermoelectric materials, the safety of the sensor during service was greatly improved; thus, the reliability of curved TFTCs could be efficiently improved.

When simulating a bending deformation, the bottom and top surfaces of the sensors were all fixed. At the center of the back of the substrate, a uniform distributed load of 1.0e6 N/m^2^ was applied in a circle with a diameter of 1 mm. The distribution of internal stress, the detailed diagram of the maximum stress force and the deformation diagram of the linear and curved sensors are displayed in Fig. 4d, e, respectively. Regarding the linear electrode, the maximum stress value appeared in the middle of the thermoelectrodes. The stress field was symmetrically distributed and gradually decreased toward the cold and thermosensitive positions. The stress field of the curved sensor was basically the same as that of the linear sensor, and its maximum stress value appeared in the middle of the whole structure; however, its maximum stress was significantly reduced compared with the linear sensor.

From the simulation, we can see that there was no effect on the thermoelectric characteristics of the sensors by adjusting the structure of the thermoelectrodes. However, by changing the linear electrodes into curved electrodes, the resistance of the sensor to deformation and damage was greatly improved. It is believed that the addition of a microscale wavy substrate, the three-dimensional structure of the sensor will have better stability for measuring surface temperature.

## Results

An orthogonal experiment was conducted to reduce the number of experiments as much as possible while ensuring the accuracy of the test results. Three factors, including the sputtering power, argon flow rate and vacuum degree, were selected according to the preparation process of the magnetron sputtering technology. In addition, three gradients were selected for each parameter for further comparison^25^. With the help of the orthogonal experiment, numerous samples were prepared to obtain an optimized set of thermoelectrode parameters (In_2_O_3_) that could obtain thin films with the maximum resistance to the influence of deformation. The parameters of the orthogonal test are shown in Table 1.

**Table 1:** Selection of the parameters of the orthogonal experiment

Parameters		Level one	Level two	Level three
A	Sputtering power (W)	100	150	200
B	Argon flow rate (sccm)	30	60	90
C	Vacuum degree (Torr)	3e−5	8e−6	3e−6

According to orthogonal experiment theory, the corresponding orthogonal table was acquired (Table 2). Two types of temperature-sensitive materials were fabricated based on the parameters in Table 2, and a total of nine samples were obtained by magnetron sputtering technology, becoming the basis for the further optimization of the experiment.

**Table 2:** Orthogonal experiment for the In_2_O_3_ thermoelectrode

Experimental number	Sputtering power (W)	Argon flow rate (sccm)	Vacuum degree (Torr)
1	100	30	3e−5
2	100	60	8e−6
3	100	90	3e−6
4	150	30	8e−6
5	150	60	3e−6
6	150	90	3e−5
7	200	30	3e−6
8	200	60	3e−5
9	200	90	8e−6

The deformation resistance of the thin films prepared under the different process parameters was determined by a bending test. The bending test methods of the thin films deposited on the surface of flexible substrates can be divided into three categories: concentric axis experiment, two-point bending experiment, and x–y–θ coordinate experiment^30–32^.

Among the three methods, the concentric axis experiment is the simplest method to characterize the bending characteristics of the samples based on the geometry of the cylinder. The specific experimental method of the concentric axis experiment is as follows: a large number of the same specimens are attached to a series of cylindrical surfaces with different radii for concentric bending. Under similar operating conditions, the degree of influence of the deformation on the specimen can be evaluated and contrasted^26^.

The advantage of the concentric axis experiment is its simple operation. The bending curve of the samples are treated with a standard circle by attaching the samples to the outer surface of cylinders. Through this method, the characteristics of the samples can be quickly and conveniently obtained, and the calculation and processing of experimental data can be simplified. The disadvantage of this method is that it only provides simple radius of curvature data while requiring a large number of experimental samples to perform a large number of bending experiments. Moreover, it cannot guarantee a continuous change in the radius of curvature from large to small. In addition, the force and strain on the entire surface of the samples after bending are approximately the same, which is different from the uneven surface load during actual use.

**Fig. 5:**
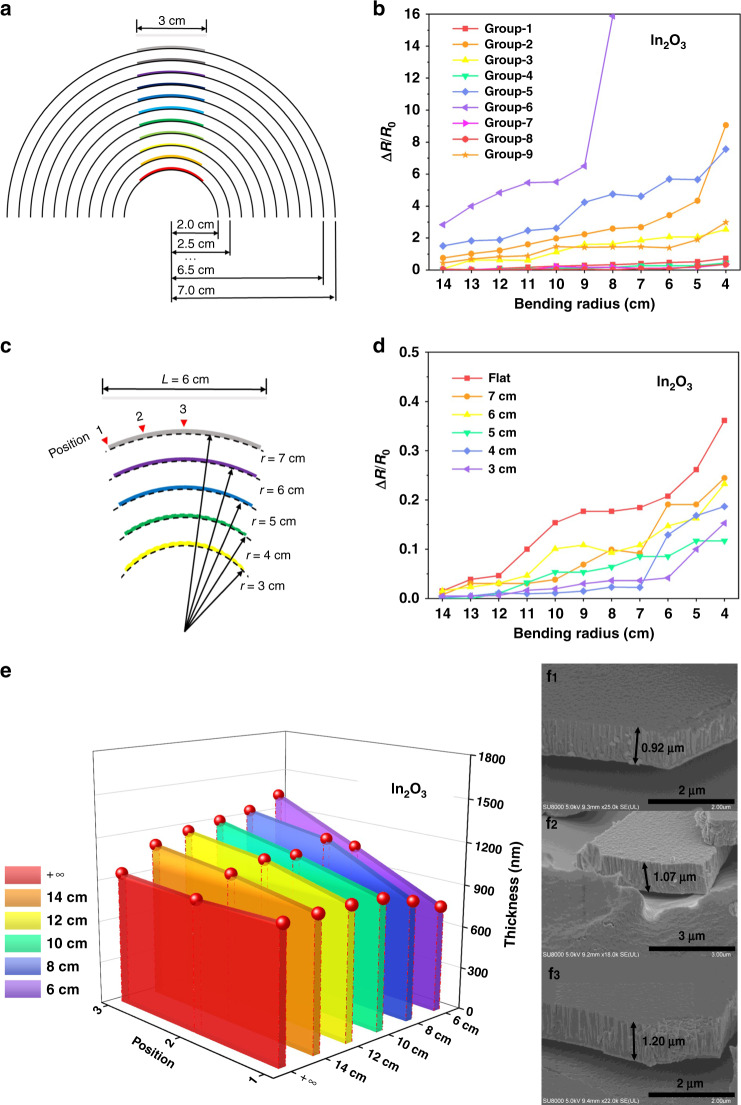
Flexible design and preparation of thermoelectrodes. **a** Thin films sequentially attached to the plane and circles with radii of 2–7â€‰cm. **b** Resistance change rates of the In_2_O_3_ thermoelectrodes attached surfaces with different curvatures. **c** Magnetron sputtering method combined with the substrate prebending method to prepare the thin films. **d** Relative resistance change rates of the In_2_O_3_ thermoelectrodes prepared on substrates with different bending curvatures. **e** Thickness measurement at three positions of the In_2_O_3_ thermoelectrodes on substrates with different degrees of prebending. **f** SEM images of the In_2_O_3_ thermoelectrodes prepared on an arc surface with a radius of curvature of 5â€‰cm: **f**_**1**_ position 1, **f**_**2**_ position 2, and **f**_**3**_ position 3.

As shown in Fig. 5a, the 9 sets of samples prepared in the experiment were cut into strips with a length of 2.5 cm and a width of 0.5 cm. The samples were attached to a circle with a radius of 2–7 cm in turn. A multimeter was used to measure the resistance at both ends along the length of the samples. The resistance of an unbent thin film in a plane was applied as the reference resistance value. The relative change in the resistance of the thin films on the surfaces with different curvatures is employed as the factor to evaluate the resistance of the film to deformation. According to the relative change in resistance in Fig. 5b, it can be seen that as the radius of the curved surface to which the thin films are attached gradually decreases (the curvature of the curved surface gradually increases), the relative change in the thin film resistors gradually increases. By comparing the test results, the seventh group of In_2_O_3_ thermoelectrodes in the orthogonal test demonstrated better initial resistance to deformation than the other samples. Notably, the maximum change of the other group exceeded 10 times the resistance. Thus, it is believed that the improper combination of magnetron sputtering parameters leads to films with poor toughness. When connected to a curved surface with a large curvature, the internal fracture of the thermoelectrode leads to a multistep increase in resistance. According to the range analysis after processing the data of the orthogonal experiment, it can be inferred that the influence of power and flow rate on the flexibility were the top two parameters. The vacuum degree had the least effect. Importantly, all samples show a tendency to become less flexible as the flow rate increased. Therefore, it is essential to select a small flow rate for the preparation of In_2_O_3_ thermoelectrodes. In addition, a higher vacuum degree is undoubtedly one of the basic requirements to ensure the quality of the prepared film. A higher sputtering power can ensure high efficiency during film preparation. The activity of particles reaching the substrate surface and the scattering degree of particles are affected not only by the power, air flow, and vacuum degree in the orthogonal experiment but also by the distance between the target and substrate, the degree of target use and other factors. In essence, all of the preparation parameters have a direct impact on the characteristics of the thermoelectrode (Supplementary material).

In 2020, Liu et al.^8^ reported that when a sensitive thin film was bent inward due to pressure, the characteristics of the thin films remained basically stable. In contrast, when the thin film was bent outwards due to tension, the characteristics of the thin films changed greatly as the degree of bending increased. To further improve the ability of thin films to resist deformation with the best process preparation parameters, an innovative method based on magnetron sputtering is proposed, that is, to bend the substrate to different degrees before preparation. In this way, if the thermoelectrode is prepared on the surface of a circle with a radius of 3 cm, when the thermoelectrode is placed on the surface of a circle with a radius of more than 6 cm for measurement and application, the thermoelectrode is equivalent to the compressed state, and the deformation has little effect on its characteristics. In this way, the whole sensor shows improved resistance to deformation. Therefore, before placing the substrate in the magnetron sputtering chamber, the flexible substrate is cut according to the sensor size (5.5 cm × 1 cm) and attached to the surface of a stainless steel plate of the same size. As illustrated in Fig. 5c, the stainless steel plates are bent according to standard circles with radii of 2–7 cm. The In_2_O_3_ thermoelectrodes prepared on substrates with different bending curvatures are then subjected to bending tests again.

As shown in Fig. 5d, based on the optimal parameters selected by the orthogonal experiment, the ability of the In_2_O_3_ thermoelectrodes to resist deformation is further strengthened by the prebending preparation method. At the same time, the maximum relative resistance change rate of the optimized In_2_O_3_ thermoelectrode is only 11.7% when fabricated on a round surface with a radius of 5 cm. However, during the preparation process, it is also observed that a greater degree of bending of the base is not better. On the one hand, this result is due to the size limitation of the sensor. On the other hand, when magnetron sputtering is used to prepare curved thin films, the uniformity of the film thickness will be seriously affected. The poor uniformity of a thin film will directly affect its mechanical properties. Because of the flexible substrate of the sensor, it is difficult to accurately measure the thickness of the thin films in traditional ways, such as step meters, atomic force microscopy and other methods. Therefore, scanning electron microscopy (SEM) was used to visually observe the cross-section of the In_2_O_3_ thermoelectrodes. The curvature of the curved surface of the substrate increases, and the uniformity of the film thickness gradually decreases. This result is due to the limitations of the manufacturing process of magnetron sputtering technology, and this situation can hardly be avoided. During the preparation process, the In_2_O_3_ particles are bombarded by argon ions, leading to movement from top to bottom in the chamber and deposition on the substrate to form a thin film. This mechanism might be the reason why the best prebending degree of the material is not the surface with the maximum curvature. A larger change in the thickness of the In_2_O_3_ thin film inhibits its ability to resist deformation. Finally, the preparation process parameters of the platinum and In_2_O_3_ thermoelectrodes of the sensor were determined, and the results are shown in Table 3. As shown in Fig. 5e, f, the thickness of the In_2_O_3_ thermoelectrodes on substrates with different degrees of prebending were measured by SEM at three positions.

**Table 3:** Optimum preparation parameters of the flexible temperature sensor

Material		Sputtering current (amps)	Argon flow rate (sccm)	Vacuum degree (Torr)	Curvature of substrate (m^−1^)
*Positive electrode*	Platinum	0.4	20	3e−5	0
**Material**		**Sputtering power (W)**	**Argon flow rate (sccm)**	**Vacuum degree (Torr)**	**Curvature of substrate (m**^**−1**^**)**
*Negative electrode*	In_2_O_3_	200	30	3e−6	10

**Fig. 6:**
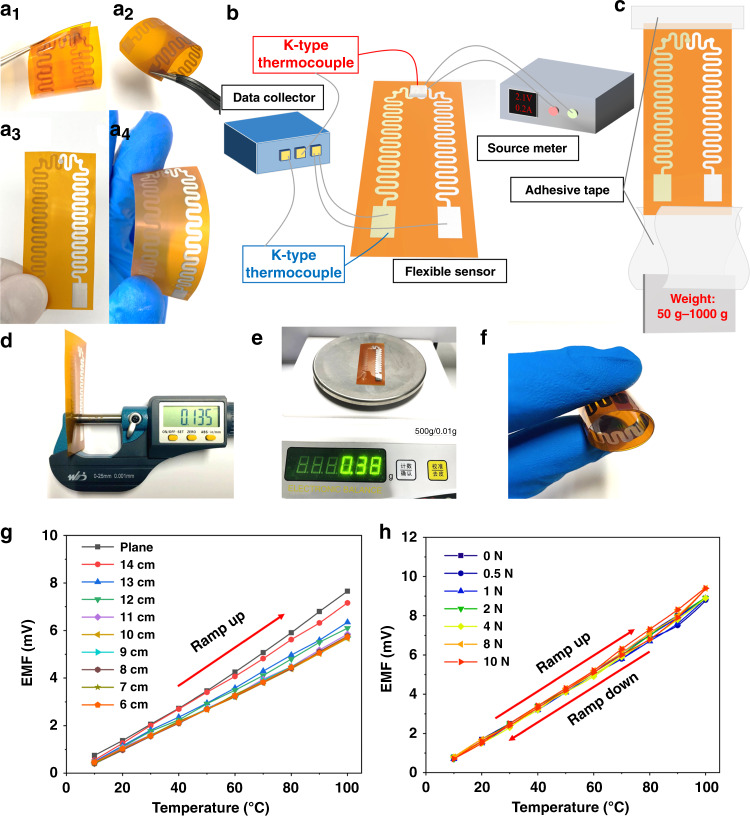
Test system and results of the flexible micro-three-dimensional temperature sensor. **a** Fabricated samples of the sensor. **b** Sensor testing system. **c** Schematic diagram showing the setup for applying tension to the sensor. **d** Ultrathin. **e** Ultralight. **f** Ultraflexible. **g** Thermoelectric output characteristics of the sensors attached to surfaces with different curvatures. **h** Service characteristics of the sensor under different tension conditions. The force of gravity exerted by the standard weight is used as a pulling force on the sensor.

A variety of flexible sensors were prepared during the experimental preparation, as shown in Fig. 6a. From the polyimide substrate of the flexible temperature sensor, it was found that the substrates of samples a_1_ and a_3_ produced specular reflection, while the diffuse reflection of light occurred with the substrates of samples a_2_ and a_4_. However, all as-prepared samples displayed excellent flexibility.

To test and calibrate the prepared sensor, the test system shown in Fig. 6b, c was constructed. The K-type thermocouples were placed at the thermosensitive position of the sensor to the temperature of the ceramic heater, which was controlled and heated by changing the voltage and electricity. The other K-type thermocouples were used to realize the real-time monitoring of the terminal of the sensor. Then the temperature of the K-type thermocouples and the electromotive force of the flexible sensors were recorded by the data collector. As shown in Fig. 6d, f, the three pictures show the ultrathin, ultralight and ultraflexible characteristics of the temperature sensor. Using the system in Fig. 6b, the static characteristics of the sensor attached to the surfaces with different curvatures were tested. From the display results (Fig. 6g), it can be seen that the sensors attached to the different surfaces have great linearity when tested. With an increasing surface curvature, the test sensitivity of the sensor decreased slightly and then remained stable, indicating that the sensor is almost unaffected by the shape of the attached objects and shows a greatly improved fault tolerance rate of the test position in actual applications. As illustrated in Fig. 5f_2_, although the preparation parameters and preparation process of the thermoelectrode are highly optimized, cracks in the In_2_O_3_ thermoelectrode during testing are still inevitable, which leads to a small change in the output characteristics. When the temperature difference between the thermosensitive position and terminal is 100 °C, the maximum sensitivity (76.5 μV/°C) of the sensor is obtained.

On the other hand, the thermoelectric characteristics of the sensor under different tensile forces were investigated. To quantify the applied force, as shown in Fig. 6c, a series of standard weights were used as the force source. In the experiment, the sensor was placed vertically on the ground, the upper end of the sensor was fixed, and the weight was fixed on the lower end surface of the sensor with adhesive tape. The sensor was again tested by the system in Fig. 6b, and the experimental results of Fig. 6h show that the overall structure exhibits excellent tensile properties. According to the simulation results in Fig. 4c, under tension of 0–10 N, it can be seen that as the tensile force increases, the maximum internal stress of the sensitive thermoelectrodes of the sensor show a positively correlated increase; however, the sensor still exhibits fewer hysteresis characteristics and maintains good stability and reliability.

**Fig. 7:**
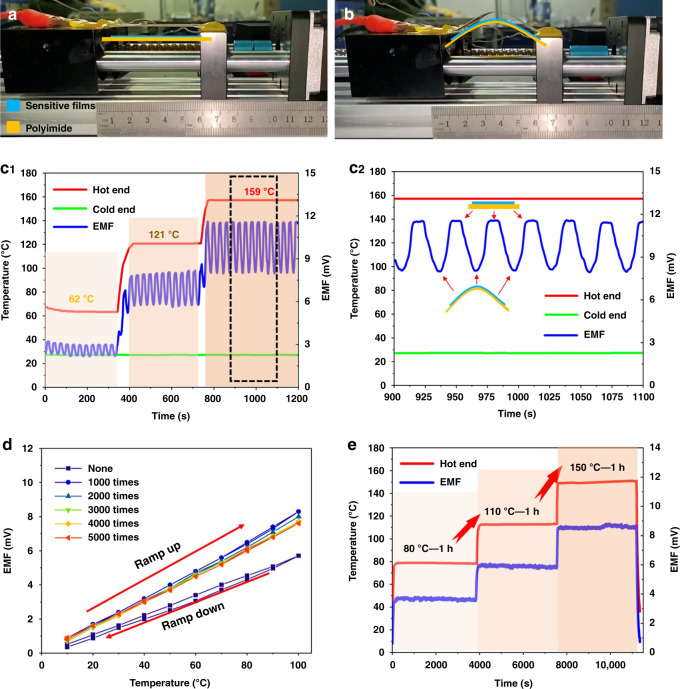
Fatigue resistance and stability test for evaluating the output characteristics of the sensor. **a** Position one, in the free state and **b** position two, in the crimped state. **c** Thermoelectric output curve of the sensor under the reciprocating motion of the lead screw. **d** Comparison of the output characteristics of the sensor after folding for a different number of times. The thermoelectric characteristic data of the sensor are collected every thousand folds, and the maximum number of folds is 5000. **e** Sensor tested at different temperatures for up to three hours. The output curve of the sensor is obtained by increasing the sensor temperature from room temperature to 150â€‰°C (in a 28–30â€‰°C ambient atmosphere).

Combined with the system in Fig. 6b, the fatigue resistance of the sensor was investigated by the lead screw. Figure 7a, b depict the two states of the sensor in the fatigue test: the natural state and maximum bending state. To further explore the characteristics of the prepared sensor, the TEMF of the flexible sensor on the reciprocating screw was continuously collected in the experiment. As shown in Fig. 7c_1_, the TEMF output of the sensor at different temperatures exhibits periodicity with its own periodic deformation. Figure 7c_2_ shows the TEMF curve of the sensor, which is the real-time signal output curve of seven reciprocating motions of the thermosensitive position of the sensor at 159 °C. With a gradual increase in the deformation of the sensor under pressure at both ends, the TEMF value at the same temperature shows a decreasing characteristic. Interestingly, with a decrease in deformation, the signal recovery is very fast, and the sensor can still achieve real-time temperature measurements. Additionally, when the sensor returns to its natural state, it can still accurately measure the temperature of the thermosensitive position and exhibits excellent temperature reproducibility and stability. The fabricated sensor with the micro-three-dimensional structure for measuring temperature delivers excellent performance similar to a spring, showing good “elasticity” and “recovery”. In addition, in Fig. 7d, the anti-fatigue characteristics of the sensor were evaluated by reciprocating a lead screw up to 5000 times. Different from the initial test results, the sensitivity of the sensor improves from 57.1 μV/°C to 83.0 μV/°C after 1000 deformation cycles and remains stable as the number of deformation repetitions increase. The reason for this situation is that the toughness of the In_2_O_3_ thermoelectrode has difficulty reaching the level of the metal material. In the process of stress deformation, many cracks gradually form in the thin film and expand as the degree of deformation increases. Although the structure is damaged to a certain extent during stress deformation, the sensor can still detect the temperature due to its optimized design. Finally, the temperature retention of the flexible sensor was evaluated, and the sensitive end of the sensor was measured at three different temperatures for insulation. It can be seen from the results in Fig. 7e that the sensor exhibits good working reliability and temperature retention and can work continuously for multiple hours. When the test temperature is 80 °C, 110 °C, and 150 °C, the temperature drift rate of the sensor is 8.60 °C/h, 5.15 °C/h and 3.99 °C/h, respectively, ensuring the measurement accuracy and stability of the sensor during service.

**Fig. 8:**
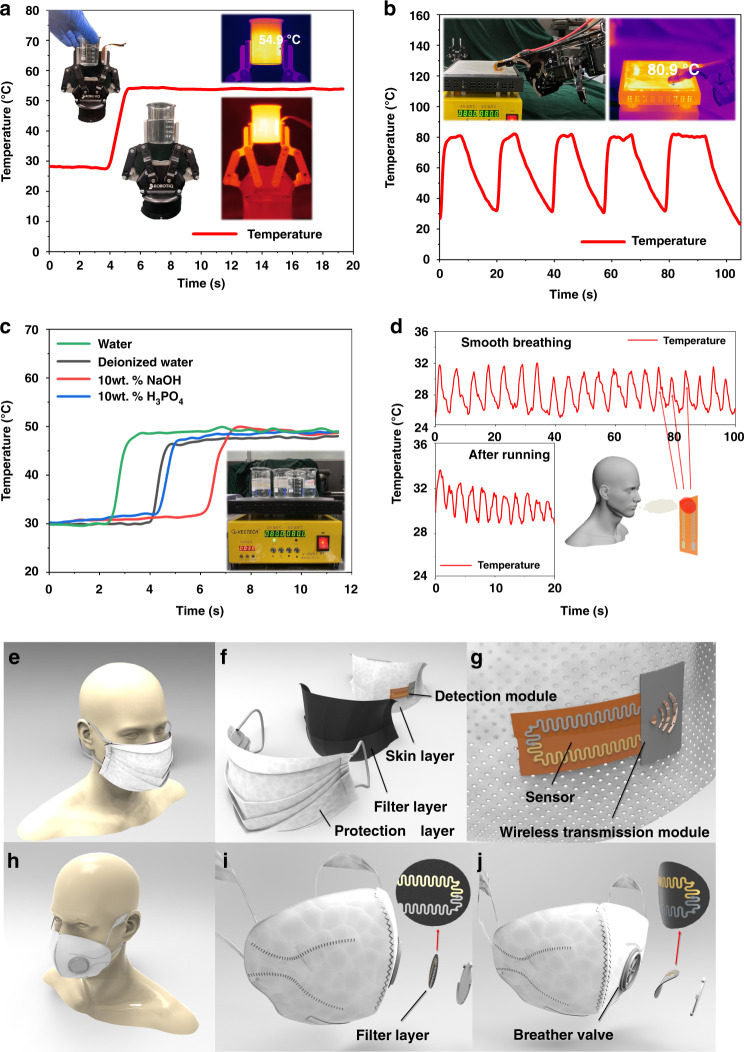
Display of sensors in some practical applications. **a** Obtaining temperature information of an object from a sensor attached to a gripping manipulator. The sensor is placed in the industrial two-finger gripper to realize the accurate monitoring of the temperature of the object. **b** Obtaining information from the environment. The sensor is attached to the tip of a smart manipulator as a bionic skin. **c** Test characteristics of sensors in different kinds of solutions. A robotic finger attached to a sensor is placed in water, deionized water, 10 wt.% NaOH and 10 wt.% H_3_PO_4_ on the same heating platform. **d** Continuous real-time monitoring of breathing (smooth breathing and breathing after running). Stable monitoring of the temperature variation generated by exhalation and inhalation (the ambient temperature is approximately 25 °C) by a fabricated temperature sensor placed in front of the mouth. Models of sensors used in two kinds of masks: **e** Nonbreathing valve mask and an **f** Exploded view of a mask. Based on the ultralight, ultrathin and ultrasoft characteristics of the sensor, the breathing monitoring unit is placed in the middle of the mask to realize the real-time measurement of breathing. **g** Detection module. Based on the wireless transmission module, the acquired information is transmitted in real time. **h** Mask with a breathing valve. **i** Detailed picture of the mask as the person inhales. **j** Detailed picture of the mask as the person exhales. The sensor is combined with a filter disc, and the excess substrate of the sensor is removed to reduce the impact on filter performance.

After that, the test of the sensor in different application scenarios was conducted. The low thickness and low rigidity of the flexible sensor allowed it to be attached to the various curvatures that randomly occurred on the facial surface of the manipulator, as shown in Fig. 8a, b. During the test, the flexible temperature sensor was placed on one side of the two-finger mechanical gripper, and the tip of the finger of the bionic manipulator was placed to clamp the beaker filled with hot water, continuously touching the thermal platform. To compare the reliability of the test data results, the temperature of the beaker and the thermal platform were visualized through the thermal imager. The temperature sensor presents a stable perception of the temperature of the beaker wall and platform. According to the thermographic images, the sensor possesses high test accuracy and good temperature retention.

To evaluate the performance of the sensor in different environments, the sensor was tested in different acids, bases, and aqueous solutions. To further eliminate the chemical stimulation of the sensor-sensitive film layer, a thin polyimide encapsulating film was added for protection. The sensor-sensitive end was then placed in the solution. Since all solutions were placed on the same heating table and reached an equilibrium state of heat exchange, the temperature of the various solutions was basically the same. According to the experimental results in Fig. 8c, the sensor effectively completes the task of measuring the temperatures of the various liquids. The steady-state temperature is nearly uniform in the different solutions, proving that the sensor can adapt to normal operation in harsh test environments (including acidic and alkali gases or acidic and alkali liquids); furthermore, this result suggests its tremendous application potential for measuring liquid temperatures in industrial pipelines. Based on the ultraflexibility of the sensor, it is believed that the noninterfering measurement of the flow field in a pipe can be realized more effectively.

Furthermore, we successfully compared the relationship between changes in respiratory characteristics and increased physical activity in Fig. 8d. Two sets of data clearly display the difference between the smooth breathing state and the breathing state after short-term activities. When the human body is in a stable state of life, it has regular breathing and even inspiratory and expiratory frequencies. However, exercise will lead to an increase in the metabolism of the human body, resulting in hyperventilation. Breathing becomes significantly faster, and the frequency increases sharply. Benefiting from the ultrathin characteristics, small heat capacity and fast response characteristics of thin films, the sensor can realize the real-time monitoring of breath temperature. Under normal circumstances, the changes in people’s breathing are an outstanding indicator of changes in emotional or physical states. Real-time monitoring of the sensor can detect changes in respiratory patterns so that custodians or data centers can quickly identify people with abnormal metabolic and pathologic dyshomeostasis caused by diseases, such as acidosis, alkalosis, and acute asthma.

The as-prepared flexible temperature sensor with great service stability and reliability has very high application prospects and value. In many fields, flexible temperature sensors have been initially applied to mechanical fingers and integrated with force sensors and material recognition sensors to be used in electronic skins^4,15,32,33^. Additionally, a flexible temperature sensor is also expected to be used for real-time in situ detection of human respiratory temperature in the future to make a certain contribution to the fight against or prevention of COVID-19. Since the sensor is a passive sensor and does not require an external power supply device, it can be combined with a wireless transmitting device and placed in a mask to detect the temperature of a person’s exhaled breath and provide real-time feedback, thereby achieving real-time monitoring of changes in human health. The flexible temperature sensor can be used repeatedly and carry information similar to a bank card. According to the presence or absence of breather valves, the masks can be divided into two types. When the flexible temperature sensor is applied to a respirator without a breathing valve, the ultrathin, ultralight and ultraflexible sensors can be placed between the inner layer and functional filter layer in the respirator to detect respiration; thus, it can be inserted into a mask to measure temperature similar to a “bank card” inserted into an automatic teller machine. It is worth mentioning that a clamping structure can be designed on the mask so that the sensor can be removed after the mask is contaminated similar to a “bank card” being pulled out of a cash machine; thus, the sensor can be reused in multiple masks. For a respirator with a breathing valve, it is desirable to prepare the sensor on the surface of the filter membrane or combine it to realize the detection of human breathing and temperature. In this way, the collection of personal health information can be greatly enhanced, allowing a database to be established to ensure the physical status of key populations and contributing to the construction of a noninvasive physiological temperature sensor monitoring system^34,35^. This sensor can also be used for special operations groups or individual workers with long shifts, such as underground miners, border guards in harsh environments, and long-distance freight drivers. The previous tests can prove that the sensor works normally under harsh conditions, thus realizing the real-time monitoring of the vital signs of the individual to ensure their personal safety. When the signal collected by the sensor finds that the breathing of the individual is weak or absent, the feedback center can be warned as soon as possible, allowing the center to immediately inquire about the situation to ensure personal safety. The application models of the sensor for masks are provided in Fig. 8e–j.

The introduction of this kind of smart mask can provide guarantees of life for people with acute illness. It can detect patients with shortness of breath or weak breathing in real time, allowing a feedback center to provide timely care and inquiries. The presence of smart masks can detect accidents in elderly individuals in real time to avoid tragedies that no one has discovered and provide a basis for the survival of lonely elderly individuals at home.

## Discussion and conclusion

A passive flexible temperature sensor with great deformation resistance, fatigue resistance, stability, and high sensitivity was successfully prepared. We expect that the flexible temperature sensor can be directly applied to an e-skin and smart manipulator or integrated with other types of sensors to verify its high potential toward the continuous monitoring of physiological information. Additionally, it can also be combined with masks to detect and evaluate the physiological health of the human body, which is an urgent demand for preventive medicine and industrial production. The timely detection of anomalies by sensors can greatly reduce economic losses and save lives. Although the proposed idea is still in its infancy, we hope that further research on this idea can make a large contribution to the development of flexible temperature sensors and in the practical application of epidermal sensors in artificial skin for physiological temperature monitoring.

## Supplementary information

Supplementary information
