# Supplementary material for: A thin-film temperature sensor based on a flexible electrode and substrate
Source: Microsyst Nanoeng. 2021 Jun 1;7:42. doi: 10.1038/s41378-021-00271-0 (PMC8166532; doi:10.1038/s41378-021-00271-0)
Supplement: Supplementary file 1 — Supplementary information [file 41378_2021_271_MOESM1_ESM.docx]

# Supplementary Information

The test data are supplemented below:

**Table 1** Selection of orthogonal experiment parameters.

| **Parameters** | | **Level One** | **Level Two** | **Level Three** |
| --- | --- | --- | --- | --- |
| **A** | Sputtering power (W) | 100 | 150 | 200 |
| **B** | Argon flow rate (sccm) | 30 | 60 | 90 |
| **C** | Vacuum degree (Torr) | 3e-5 | 8e-6 | 3e-6 |

**Table 2** Orthogonal experiment for In_2_O_3_ thermo-electrode.

| **Experimental number** | **Sputtering power (W)** | **Argon flow rate (sccm)** | **Vacuum degree (Torr)** |
| --- | --- | --- | --- |
| **1** | 100 | 30 | 3e-5 |
| **2** | 100 | 60 | 8e-6 |
| **3** | 100 | 90 | 3e-6 |
| **4** | 150 | 30 | 8e-6 |
| **5** | 150 | 60 | 3e-6 |
| **6** | 150 | 90 | 3e-5 |
| **7** | 200 | 30 | 3e-6 |
| **8** | 200 | 60 | 3e-5 |
| **9** | 200 | 90 | 8e-6 |

**Table 3** The First Time- Resistance(kΩ) tests of indium oxide (In_2_O_3_) samples on the surfaces of circles with different curvature radii (2cm-7cm).

| **Group** | **1** | **2** | **3** | **4** | **5** | **6** | **7** | **8** | **9** |
| --- | --- | --- | --- | --- | --- | --- | --- | --- | --- |
| **Flat(R_0_)** | 3.36 | 0.905 | 2.87 | 0.267 | 0.122 | 1.09 | 0.130 | 0.120 | 0.881 |
| **7cm** | 3.36 | 1.59 | 3.08 | 0.267 | 0.306 | 4.19 | 0.132 | 0.127 | 1.270 |
| **6.5cm** | 3.41 | 1.83 | 4.66 | 0.269 | 0.345 | 5.46 | 0.135 | 0.122 | 1.490 |
| **6cm** | 3.71 | 2.01 | 4.66 | 0.280 | 0.352 | 6.38 | 0.136 | 0.120 | 1.620 |
| **5.5cm** | 3.96 | 2.35 | 4.61 | 0.290 | 0.423 | 7.06 | 0.143 | 0.128 | 1.670 |
| **5cm** | 4.18 | 2.69 | 6.09 | 0.300 | 0.441 | 7.11 | 0.15 | 0.125 | 2.170 |
| **4.5cm** | 4.33 | 2.93 | 7.45 | 0.300 | 0.639 | 8.20 | 0.153 | 0.127 | 2.130 |
| **4cm** | 4.48 | 3.25 | 7.53 | 0.311 | 0.701 | 18.5 | 0.153 | 0.122 | 2.150 |
| **3.5cm** | 4.70 | 3.33 | 8.21 | 0.340 | 0.685 | 23.6 | 0.154 | 0.131 | 2.160 |
| **3cm** | 4.95 | 4.01 | 8.81 | 0.340 | 0.816 | - | 0.157 | 0.129 | 2.100 |
| **2.5cm** | 5.09 | 4.83 | 8.80 | 0.340 | 0.812 | - | 0.164 | 0.149 | 2.550 |
| **2cm** | 5.80 | 9.10 | 10.2 | 0.387 | 1.05 | - | 0.177 | 0.163 | 3.510 |

**Table 4** The First Time- Resistance(kΩ) variation of In_2_O_3_ sample on the surface of a circle with different radius (2cm-7cm).

| **Variation (R)** | **1** | **2** | **3** | **4** | **5** | **6** | **7** | **8** | **9** |
| --- | --- | --- | --- | --- | --- | --- | --- | --- | --- |
| **7cm** | 0.00 | 0.685 | 0.210 | 0.000 | 0.184 | 3.10 | 0.002 | 0.007 | 0.389 |
| **6.5cm** | 0.05 | 0.925 | 1.79 | 0.002 | 0.223 | 4.37 | 0.005 | 0.002 | 0.609 |
| **6cm** | 0.35 | 1.10 | 1.79 | 0.013 | 0.230 | 5.29 | 0.006 | 0.000 | 0.739 |
| **5.5cm** | 0.60 | 1.45 | 1.74 | 0.023 | 0.301 | 5.97 | 0.013 | 0.008 | 0.789 |
| **5cm** | 0.82 | 1.79 | 3.22 | 0.033 | 0.319 | 6.02 | 0.020 | 0.005 | 1.29 |
| **4.5cm** | 0.97 | 2.03 | 4.58 | 0.033 | 0.517 | 7.11 | 0.023 | 0.007 | 1.25 |
| **4cm** | 1.12 | 2.35 | 4.66 | 0.044 | 0.579 | 17.4 | 0.023 | 0.002 | 1.27 |
| **3.5cm** | 1.34 | 2.43 | 5.34 | 0.073 | 0.563 | 22.6 | 0.024 | 0.011 | 1.28 |
| **3cm** | 1.59 | 3.11 | 5.94 | 0.073 | 0.694 | - | 0.027 | 0.009 | 1.22 |
| **2.5cm** | 1.73 | 3.93 | 5.93 | 0.073 | 0.690 | - | 0.034 | 0.029 | 1.67 |
| **2cm** | 2.44 | 8.20 | 7.29 | 0.120 | 0.923 | - | 0.047 | 0.043 | 2.63 |

**Table 5** The First Time- Resistance variation/ Initial resistance of In_2_O_3_ sample on the surface of a circle with different radius (2cm-7cm).

| **Variation /Initial resistance** | **1** | **2** | **3** | **4** | **5** | **6** | **7** | **8** | **9** |
| --- | --- | --- | --- | --- | --- | --- | --- | --- | --- |
| **7cm** | 0.00% | 75.69% | 7.32% | 0.00% | 150.82% | 283.35% | 1.54% | 5.83% | 44.15% |
| **6.5cm** | 1.49% | 102.21% | 62.37% | 0.75% | 182.79% | 399.54% | 3.85% | 1.67% | 69.13% |
| **6cm** | 10.42% | 122.10% | 62.37% | 4.87% | 188.52% | 483.71% | 4.62% | 0.00% | 83.88% |
| **5.5cm** | 17.86% | 159.67% | 60.63% | 8.61% | 246.72% | 545.93% | 10.00% | 6.67% | 89.56% |
| **5cm** | 24.40% | 197.24% | 112.20% | 12.36% | 261.48% | 550.50% | 15.38% | 4.17% | 146.31% |
| **4.5cm** | 28.87% | 223.76% | 159.58% | 12.36% | 423.77% | 650.23% | 17.69% | 5.83% | 141.77% |
| **4cm** | 33.33% | 259.12% | 162.37% | 16.48% | 474.59% | 1588.01% | 17.69% | 1.67% | 144.04% |
| **3.5cm** | 39.88% | 267.96% | 186.06% | 27.34% | 461.48% | 2063.77% | 18.46% | 9.17% | 145.18% |
| **3cm** | 47.32% | 343.09% | 206.97% | 27.34% | 568.85% | - | 20.77% | 7.50% | 138.37% |
| **2.5cm** | 51.49% | 433.70% | 206.62% | 27.34% | 565.57% | - | 26.15% | 24.17% | 189.44% |
| **2cm** | 72.62% | 905.52% | 254.01% | 44.94% | 756.56% | - | 36.15% | 35.83% | 298.41% |

**Table 6** The First Time- Range analysis of data.

| **Deposition rate** | **Sputtering power** | **Argon flow rate** | **Vacuum degree** |
| --- | --- | --- | --- |
| k_1_ | 410.72% | 51.24% | 724.07% |
| k_2_ | 955.09% | 565.97% | 416.29% |
| k_3_ | 123.47% | 872.06% | 348.91% |
| R | 831.62% | 820.82% | 375.17% |
| Rank | **1** | **2** | **3** |

**Table 7** The Second Time- Resistance(kΩ) tests of indium oxide (In_2_O_3_) samples on the surfaces of circles with different curvature radii (2cm-7cm).

| **Group** | **1** | **2** | **3** | **4** | **5** | **6** | **7** | **8** | 9 |
| --- | --- | --- | --- | --- | --- | --- | --- | --- | --- |
| **Flat(R_0_)** | 0.228 | 0.172 | 25.2 | 0.277 | 0.090 | 0.360 | 0.0510 | 0.097 | 0.235 |
| **7cm** | 0.259 | 0.307 | 36.4 | 0.283 | 0.110 | 1.19 | 0.0530 | 0.104 | 0.675 |
| **6.5cm** | 0.265 | 0.351 | 41.2 | 0.283 | 0.111 | 1.25 | 0.0533 | 0.104 | 0.680 |
| **6cm** | 0.275 | 0.480 | 80.1 | 0.291 | 0.111 | 1.33 | 0.0533 | 0.105 | 0.694 |
| **5.5cm** | 0.282 | 0.509 | 106 | 0.292 | 0.121 | 1.44 | 0.0535 | 0.108 | 0.807 |
| **5cm** | 0.286 | 0.681 | 121 | 0.300 | 0.124 | 1.50 | 0.0537 | 0.109 | 1.15 |
| **4.5cm** | 0.299 | 0.839 | 137 | 0.302 | 0.125 | 1.55 | 0.0549 | 0.111 | 1.49 |
| **4cm** | 0.302 | 0.929 | 360 | 0.308 | 0.130 | 1.62 | 0.0552 | 0.119 | 1.68 |
| **3.5cm** | 0.308 | 1.05 | - | 0.318 | 0.153 | 1.68 | 0.0560 | 0.121 | 1.73 |
| **3cm** | 0.315 | 1.43 | - | 0.319 | 0.173 | 1.88 | 0.0571 | 0.132 | 1.92 |
| **2.5cm** | 0.325 | 1.70 | - | 0.319 | 0.184 | 1.92 | 0.0579 | 0.139 | 2.11 |
| **2cm** | 0.581 | 2.06 | - | 0.322 | 0.211 | 1.98 | 0.0610 | 0.191 | 2.42 |

**Table 8** The Second Time- Resistance(kΩ) variation of In_2_O_3_ sample on the surface of a circle with different radius (2cm-7cm).

| **Variation**  **(R)** | **1** | **2** | **3** | **4** | **5** | **6** | **7** | **8** | **9** |
| --- | --- | --- | --- | --- | --- | --- | --- | --- | --- |
| **7cm** | 0.031 | 0.135 | 11.2 | 0.006 | 0.020 | 0.828 | 0.002 | 0.007 | 0.440 |
| **6.5cm** | 0.037 | 0.179 | 16.0 | 0.006 | 0.001 | 0.888 | 0.002 | 0.007 | 0.445 |
| **6cm** | 0.047 | 0.308 | 54.9 | 0.014 | 0.000 | 0.968 | 0.002 | 0.008 | 0.459 |
| **5.5cm** | 0.054 | 0.337 | 81.4 | 0.015 | 0.010 | 1.08 | 0.003 | 0.011 | 0.572 |
| **5cm** | 0.058 | 0.509 | 96.2 | 0.023 | 0.003 | 1.14 | 0.003 | 0.012 | 0.915 |
| **4.5cm** | 0.071 | 0.667 | 111.9 | 0.025 | 0.001 | 1.19 | 0.004 | 0.014 | 1.26 |
| **4cm** | 0.074 | 0.757 | 334.8 | 0.031 | 0.005 | 1.26 | 0.004 | 0.022 | 1.45 |
| **3.5cm** | 0.080 | 0.878 | - | 0.041 | 0.023 | 1.32 | 0.005 | 0.024 | 1.50 |
| **3cm** | 0.087 | 1.26 | - | 0.042 | 0.020 | 1.52 | 0.006 | 0.035 | 1.69 |
| **2.5cm** | 0.097 | 1.53 | - | 0.042 | 0.011 | 1.56 | 0.007 | 0.042 | 1.88 |
| **2cm** | 0.353 | 1.89 | - | 0.045 | 0.027 | 1.62 | 0.010 | 0.094 | 2.19 |

**Table 9** The Second Time- Resistance variation/ Initial resistance of In_2_O_3_ sample on the surface of a circle with different radius (2cm-7cm).

| **Variation /Initial resistance** | **1** | **2** | **3** | **4** | **5** | **6** | **7** | **8** | **9** |
| --- | --- | --- | --- | --- | --- | --- | --- | --- | --- |
| **7cm** | 13.60% | 78.49% | 44.44% | 2.17% | 22.22% | 228.73% | 3.92% | 7.00% | 187.23% |
| **6.5cm** | 16.23% | 104.07% | 63.49% | 2.17% | 1.11% | 245.30% | 4.51% | 7.00% | 189.36% |
| **6cm** | 20.61% | 179.07% | 217.86% | 5.05% | 0.00% | 267.40% | 4.51% | 8.02% | 195.32% |
| **5.5cm** | 23.68% | 195.93% | 323.02% | 5.42% | 11.11% | 297.79% | 4.90% | 11.11% | 243.40% |
| **5cm** | 25.44% | 295.93% | 381.75% | 8.30% | 3.33% | 314.36% | 5.29% | 12.14% | 389.36% |
| **4.5cm** | 31.14% | 387.79% | 444.05% | 9.03% | 1.11% | 328.18% | 7.65% | 14.20% | 534.04% |
| **4cm** | 32.46% | 440.12% | 1328.57% | 11.19% | 5.56% | 347.51% | 8.24% | 22.43% | 614.89% |
| **3.5cm** | 35.09% | 510.47% | - | 14.80% | 25.56% | 364.09% | 9.80% | 24.49% | 636.17% |
| **3cm** | 38.16% | 731.40% | - | 15.16% | 22.22% | 419.34% | 11.96% | 35.80% | 717.02% |
| **2.5cm** | 42.54% | 888.37% | - | 15.16% | 12.22% | 430.39% | 13.53% | 43.00% | 797.87% |
| **2cm** | 154.82% | 1097.67% | - | 16.25% | 30.00% | 446.96% | 18.82% | 96.50% | 929.79% |

**Table 10** The Second Time- Range analysis of data.

| **Deposition rate** | **Sputtering power** | **Argon flow rate** | **Vacuum degree** |
| --- | --- | --- | --- |
| k_1_ | 860.36% | 63.30% | 232.76% |
| k_2_ | 164.40% | 408.06% | 681.24% |
| k_3_ | 348.37% | 901.77% | 459.13% |
| R | 695.95% | 838.48% | 448.47% |
| Rank | **2** | **1** | **3** |

**Table 11** The First Time- Resistance(Ω) tests of indium oxide (In_2_O_3_) samples prepared on different pre-bent substrates (3cm-7cm).

| **Pre-bending radius(cm)** | **7** | **6** | **5** | **4** | **3** |
| --- | --- | --- | --- | --- | --- |
| **Flat(R_0_)** | 131.0 | 129.0 | 94.0 | 133.2 | 96.1 |
| **7cm** | 132.0 | 131.0 | 94.0 | 133.4 | 96.6 |
| **6.5cm** | 135.0 | 132.0 | 94.0 | 133.9 | 96.6 |
| **6cm** | 135.0 | 133.0 | 95.0 | 134.7 | 96.7 |
| **5.5cm** | 135.0 | 135.0 | 97.0 | 134.5 | 97.7 |
| **5cm** | 136.0 | 142.0 | 99.0 | 134.7 | 98.0 |
| **4.5cm** | 140.0 | 143.0 | 99.0 | 135.2 | 99.0 |
| **4cm** | 144.0 | 141.0 | 100.0 | 136.3 | 99.6 |
| **3.5cm** | 143.0 | 143.0 | 102.0 | 136.2 | 99.6 |
| **3cm** | 156.0 | 148.0 | 102.0 | 150.4 | 100.1 |
| **2.5cm** | 156.0 | 150.0 | 105.0 | 155.6 | 105.7 |
| **2cm** | 163.0 | 159.0 | 105.0 | 158.1 | 110.8 |

**Table 12** The Second Time- Resistance(Ω) tests of indium oxide (In_2_O_3_) samples prepared on different pre-bent substrates (3cm-7cm).

| **Pre-bending radius(cm)** | **7** | **6** | **5** | **4** | **3** |
| --- | --- | --- | --- | --- | --- |
| **Flat(R_0_)** | 54.4 | 107.4 | 64.2 | 74.7 | 56.9 |
| **7cm** | 54.5 | 108.1 | 64.4 | 75.7 | 57.4 |
| **6.5cm** | 56.4 | 107.5 | 64.5 | 76.0 | 58.4 |
| **6cm** | 57.8 | 111.3 | 64.5 | 76.2 | 59.4 |
| **5.5cm** | 58.4 | 112.0 | 64.6 | 79.9 | 59.0 |
| **5cm** | 58.8 | 112.4 | 64.6 | 80.2 | 61.8 |
| **4.5cm** | 58.5 | 115.5 | 64.7 | 86.2 | 63.0 |
| **4cm** | 58.7 | 115.7 | 64.8 | 90.4 | 63.8 |
| **3.5cm** | 59.9 | 115.4 | 65.5 | 95.9 | 70.0 |
| **3cm** | 61.9 | 117.3 | 66.2 | 101.1 | 73.3 |
| **2.5cm** | 62.8 | 117.2 | 68.5 | 103.2 | 74.4 |
| **2cm** | 65.2 | 119.2 | 68.5 | 112.6 | 78.2 |

**Table 13** The First Time- Resistance(Ω) variation of indium oxide (In_2_O_3_) samples prepared on different pre-bent substrates (3cm-7cm).

| **Variation/Initial resistance** | **7cm** | **6cm** | **5cm** | **4cm** | **3cm** |
| --- | --- | --- | --- | --- | --- |
| **7cm** | 0.76% | 1.55% | 0.00% | 0.15% | 0.52% |
| **6.5cm** | 3.05% | 2.33% | 0.00% | 0.53% | 0.52% |
| **6cm** | 3.05% | 3.10% | 1.06% | 1.13% | 0.62% |
| **5.5cm** | 3.05% | 4.65% | 3.19% | 0.98% | 1.66% |
| **5cm** | 3.82% | 10.08% | 5.32% | 1.13% | 1.98% |
| **4.5cm** | 6.87% | 10.85% | 5.32% | 1.50% | 3.02% |
| **4cm** | 9.92% | 9.30% | 6.38% | 2.33% | 3.64% |
| **3.5cm** | 9.16% | 10.85% | 8.51% | 2.25% | 3.64% |
| **3cm** | 19.08% | 14.73% | 8.51% | 12.91% | 4.16% |
| **2.5cm** | 19.08% | 16.28% | 11.70% | 16.82% | 9.99% |
| **2cm** | 24.43% | 23.26% | 11.70% | 18.69% | 15.30% |

**Table 14** The Second Time- Resistance(Ω) variation of indium oxide (In_2_O_3_) samples prepared on different pre-bent substrates (3cm-7cm).

| **Variation/Initial resistance** | **7cm** | **6cm** | **5cm** | **4cm** | **3cm** |
| --- | --- | --- | --- | --- | --- |
| **7cm** | 0.18% | 0.65% | 0.31% | 1.34% | 0.88% |
| **6.5cm** | 3.68% | 0.09% | 0.47% | 1.74% | 2.64% |
| **6cm** | 6.25% | 3.63% | 0.47% | 2.01% | 4.39% |
| **5.5cm** | 7.35% | 4.28% | 0.62% | 6.96% | 3.69% |
| **5cm** | 8.09% | 4.66% | 0.62% | 7.36% | 8.61% |
| **4.5cm** | 7.54% | 7.54% | 0.78% | 15.39% | 10.72% |
| **4cm** | 7.90% | 7.73% | 0.93% | 21.02% | 12.13% |
| **3.5cm** | 10.11% | 7.45% | 2.02% | 28.38% | 23.02% |
| **3cm** | 13.79% | 9.22% | 3.12% | 35.34% | 28.82% |
| **2.5cm** | 15.44% | 9.12% | 6.70% | 38.15% | 30.76% |
| **2cm** | 19.85% | 10.99% | 6.70% | 50.74% | 37.43% |

Therefore, based on the values measured in Table 5 and Table 9, a range analysis is conducted of the results. The *k_i_* value is on behalf of the average data of one factor at the level *i (1,2,3)*. The value of *R* represents the extent of impact which is equal to the difference between the maximum and minimum *k_i_* of single factor. The relevant results are calculated and listed in Table 6 and Table 10.

From the range analysis of the two groups of experiments, it can be seen that the ranking of influencing factors is slightly different. However, it can still be inferred that the influence of power and flow rate on the flexibility occupies the top two. Vacuum degree has less effect. Importantly, both groups of samples showed a tendency to become less flexible as the flow rate increased. Therefore, it is essential to select a small flow rate for the preparation of thin films. Higher vacuum degree is undoubtedly one of the basic requirements to ensure the quality of the film preparation, so the higher vacuum degree is selected. The higher sputtering power can ensure the high efficiency of the film preparation. Therefore, sputtering power-200W, argon flow rate-30sccm, vacuum degree-3e-6Torr are selected as the preparation parameters of the In_2_O_3_ thermo-electrode.
